# Supplementary material for: Structural Identification of Individual Helical Amyloid Filaments by Integration of Cryo-Electron Microscopy-Derived Maps in Comparative Morphometric Atomic Force Microscopy Image Analysis
Source: J Mol Biol. 2022 Apr 15;434(7):167466. doi: 10.1016/j.jmb.2022.167466 (PMC9005780; doi:10.1016/j.jmb.2022.167466)
Supplement: Supplementary data 1 [file mmc2.docx]

**SUPPLEMENTARY FIGURES**


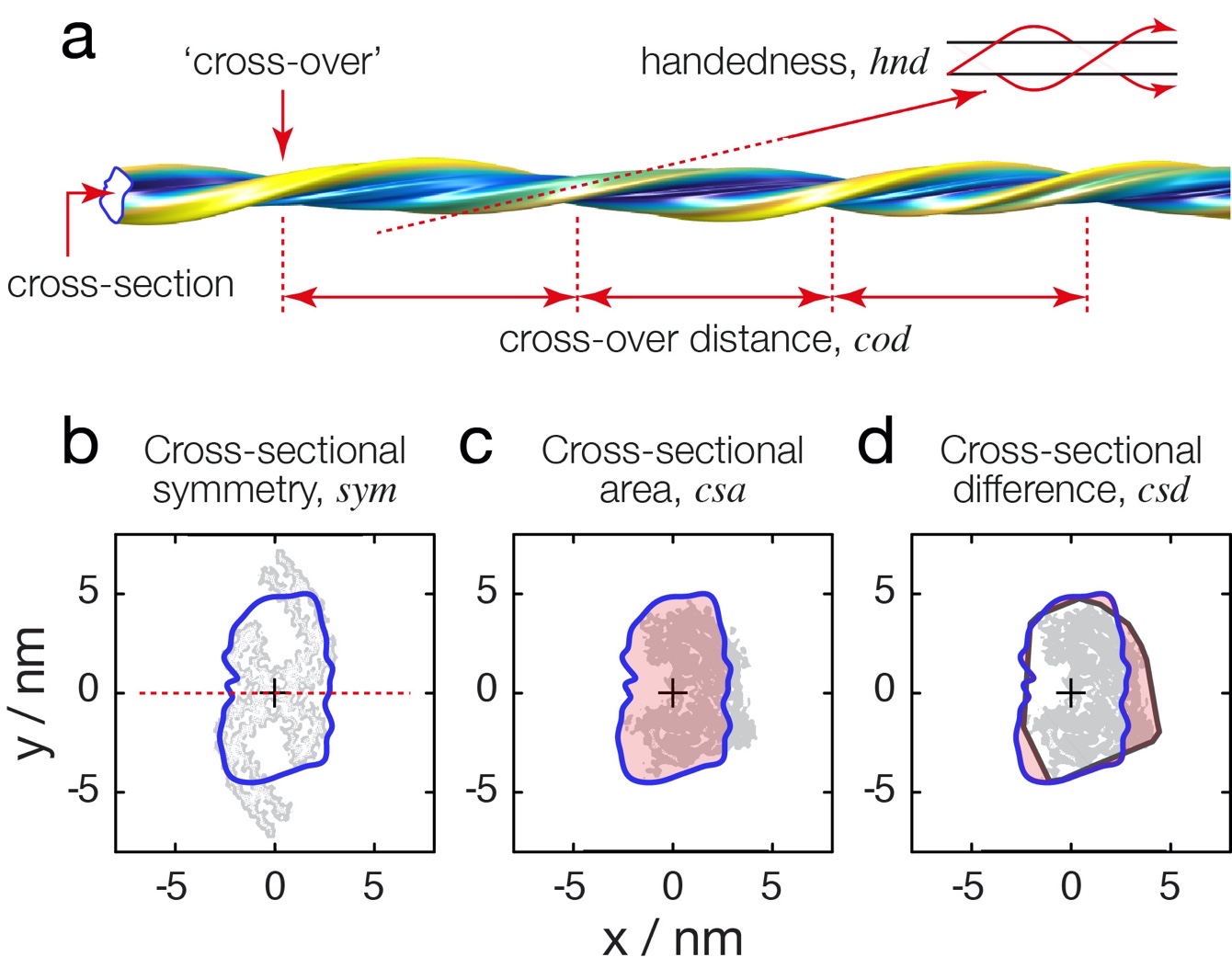


**Figure S1**. Schematic illustrations of the definitions of the morphometric parameters used for quantitative comparative structural analysis. (a) Schematic diagram illustrating the structural properties analysed, exemplified using a segment of the AFM derived dGAE fibril model shown in **Fig 1c**. The comparative morphometric parameters calculated by analysis of the cross-section are illustrated by the red line or areas in (b) for symmetry, (c) for cross-sectional area and (d) for cross-sectional difference area. Grey shapes show the cross-sections of cryo-EM density maps and the black line in (d) shows the tip-accessible cross-section, found by tracing a circle with the radius equivalent to that of the AFM tip radius around the cryo-EM density cross-section.


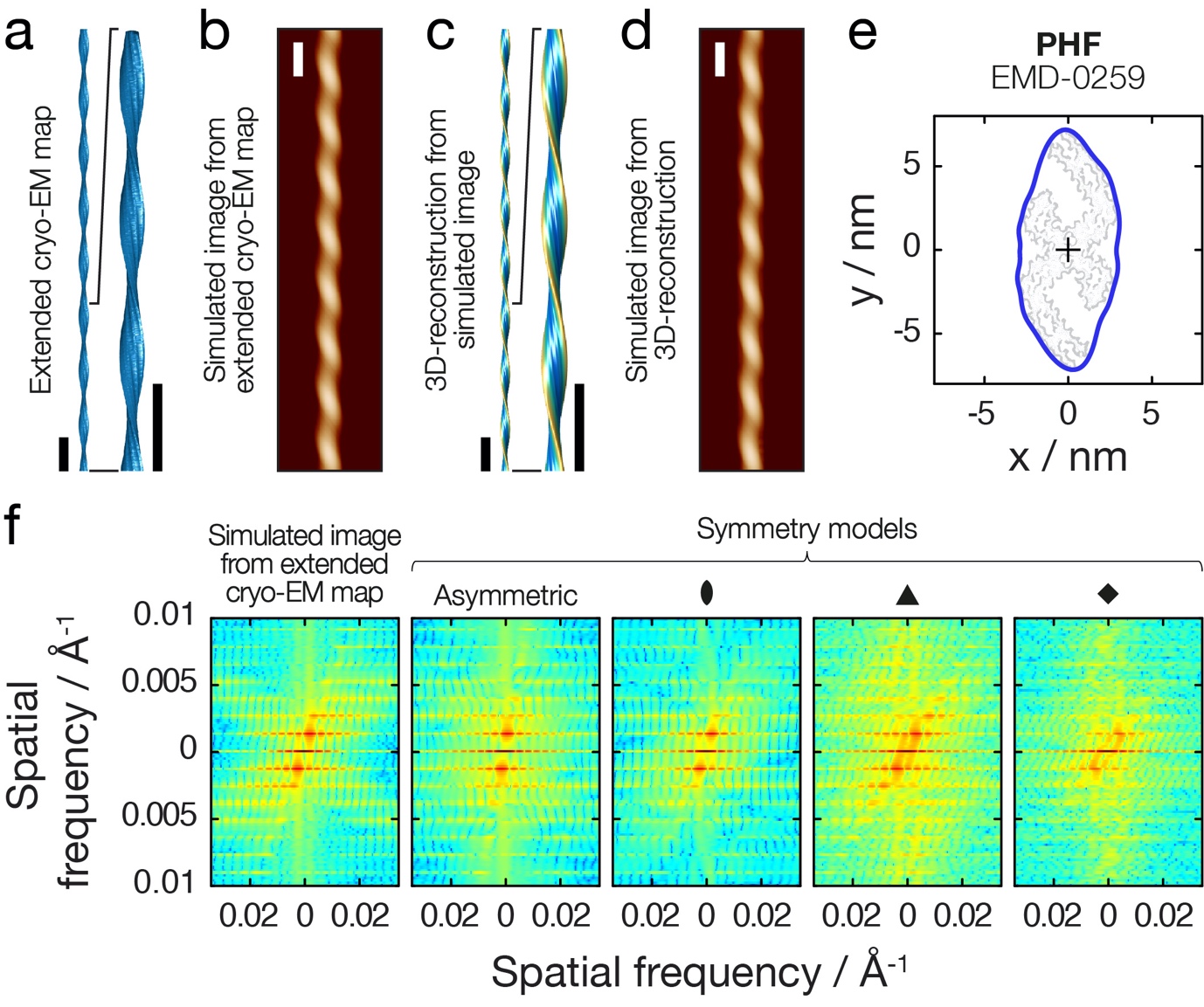


**Figure S2**. Validation of the 3D reconstruction and topograph simulation algorithms and workflow using a cryo-EM density map of PHF (EMD-0259). (a) Filament axis-aligned and lengthened cryo-EM density map of PHF. (b) Topographic AFM height image simulated from the density map in (a). (c) 3D surface envelope model reconstructed using the simulated image in (b). (d) Topographic AFM height image simulated from the 3D reconstructed model in (c). All scale bars represent 50 nm. (e) Comparison of the cross-sections of the EM density map (grey) and the AFM derived 3D model (blue). (f) Symmetry estimation during the 3D reconstruction process. As the symmetry is unknown, 3D reconstruction is performed with various symmetry estimates on the same AFM image data (here showing asymmetric, 2, 3, and 4-fold helical symmetries) and the 2D power spectra of the simulated topograph is compared to that of the original image (i.e. image shown in b). Here 2-fold symmetry gave the best match and was used for the final reconstruction.


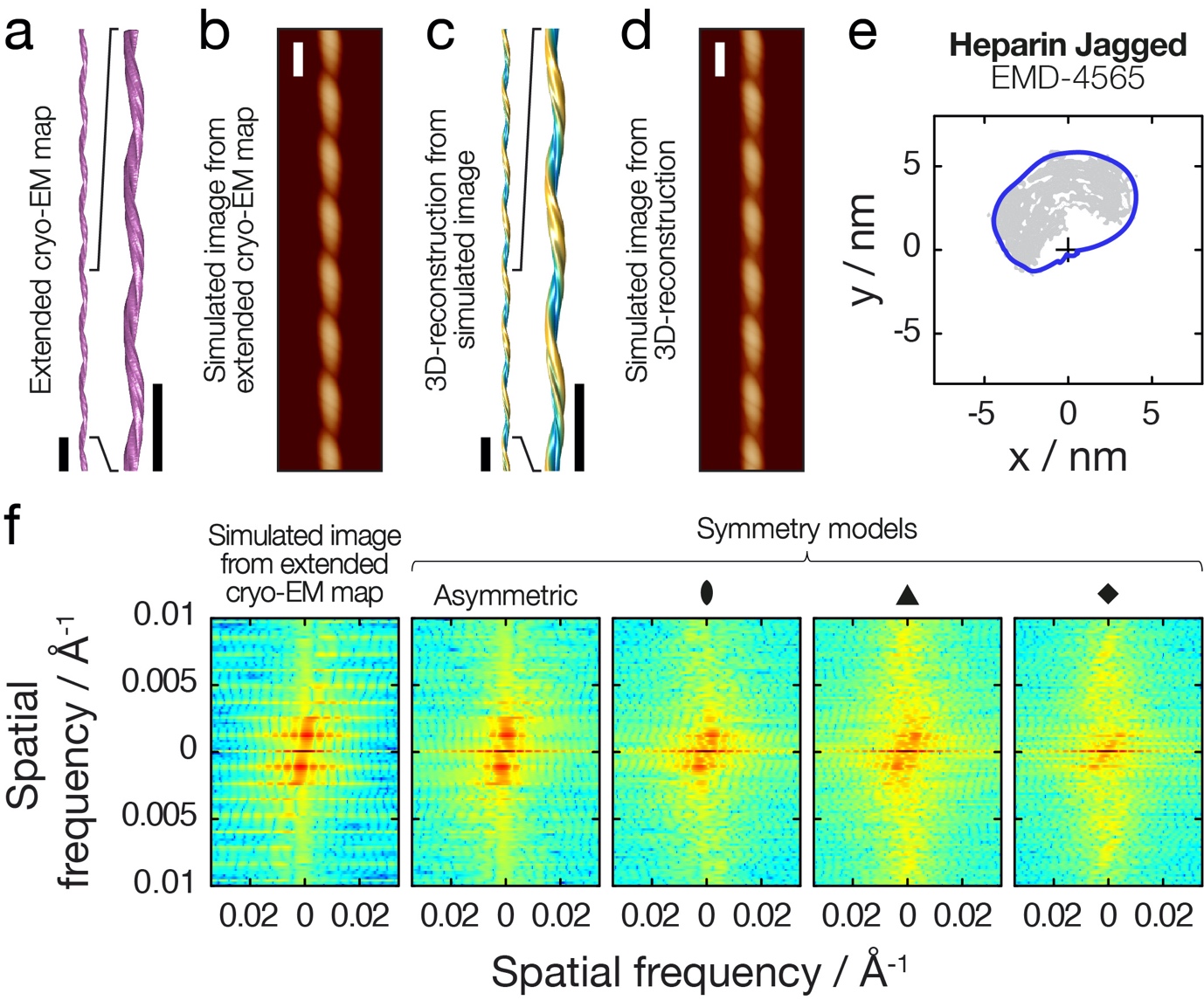


**Figure S3**. Validation of the 3D reconstruction and topograph simulation algorithms and workflow using a cryo-EM density map of a ‘heparin-jagged’ filament (EMD-4565). (a) Filament axis-aligned and lengthened cryo-EM density map of ‘heparin-jagged’. (b) Topographic AFM height image simulated from the density map in (a). (c) 3D surface envelope model reconstructed using the simulated image in (b). (d) Topographic AFM height image simulated from the 3D reconstructed model in (c). All scale bars represent 50 nm. (e) Comparison of the cross-sections of the EM density map (grey) and the AFM derived 3D model (blue). (f) Symmetry estimation during the 3D reconstruction process. As the symmetry is unknown, 3D reconstruction is performed with various symmetry estimates on the same AFM image data (here showing asymmetric, 2, 3, and 4-fold helical symmetries) and the 2D power spectra of the simulated topograph is compared to that of the original image (i.e. image shown in b). Here the asymmetric model gave the best match and was used for the final reconstruction

**SUPPLEMENTARY MOVIE**

**Movie S1**. Illustrative movie demonstrating the 3D reconstruction, structural modelling and comparative structural analysis workflow.

**SUPPLEMENTARY TABLE**

**Table S1.** Quantitative comparison of image similarity and similarity in morphometric parameters between AFM image data of the dGAE fibril shown in **Fig 1c** and cryo-EM derived tau fibril maps using the distance measure-based scoring system described in the Methods section. The similarity rank is based on the combined score *d_∑_*, from most similar with lowest *d_∑_* value to least similar with highest *d_∑_* value.

| *Filament type* | *sym* | *hnd^§^* | *cod* / nm | *csa* / nm^2^ | *csd* / nm^2^ | *d_img_* | *d_sym_* | *d_hnd_* | *d_cod_* | *d_csa_* | *d_csd_* | *d_∑_* | Rank |
| --- | --- | --- | --- | --- | --- | --- | --- | --- | --- | --- | --- | --- | --- |
| dGAE, AFM data | 2 | -1 | 49.1^†^ | 44.1 | 0.0 |  |  |  |  |  |  |  |  |
| CBD wide, EMD-10514 | 2 | -1 | 141.2 | 106.1 | 62.8 | 0.69 | 0 | 0 | 0.51 | 0.82 | 0.80 | 2.82 | 7 |
| CBD narrow, EMD-10512 | 1 | -1 | 203.9 | 54.8 | 24.3 | 0.70 | 1 | 0 | 0.86 | 0.14 | 0.31 | 3.01 | 10 |
| CTE type I, EMD-0527 | 2 | -1 | 71.1 | 83.5 | 40.2 | 0.41 | 0 | 0 | 0.12 | 0.52 | 0.51 | 1.57 | 3 |
| CTE type II, EMD-0528 | 2 | -1 | 71.1 | 64.4 | 21.3 | 0.25 | 0 | 0 | 0.12 | 0.27 | 0.27 | 0.91 | 2 |
| Narrow Pick’s filament (NPF) , EMD-0077 | 1 | -1 | 229.4 | 39.6 | 11.5 | 0.62 | 1 | 0 | 1.00 | 0.06 | 0.15 | 2.83 | 8 |
| Wide Pick’s filament (WPF) , EMD-0078 | 2 | -1 | 141.0 | 119.9 | 78.2 | 0.77 | 0 | 0 | 0.51 | 1.00 | 1.00 | 3.28 | 12 |
| Paired helical filament (PHF) , EMD-0259 | 2 | -1 | 77.6 | 61.3 | 18.4 | 0.26 | 0 | 0 | 0.16 | 0.23 | 0.23 | 0.88 | 1 |
| Straight filament (SF) , EMD-0260 | 1 | -1 | 164.8 | 70.5 | 27.9 | 0.37 | 1 | 0 | 0.64 | 0.35 | 0.36 | 2.71 | 6 |
| Heparin snake, EMD-4563 | 1 | -1 | 134.3 | 48.0 | 52.2 | 0.75 | 1 | 0 | 0.47 | 0.05 | 0.67 | 2.94 | 9 |
| Heparin twister, EMD-4564 | 1 | -1 | 50.1 | 37.3 | 12.9 | 0.46 | 1 | 0 | 0.01 | 0.09 | 0.16 | 1.72 | 4 |
| Heparin jagged, EMD-4565 | 1 | -1 | 83.3 | 45.2 | 38.8 | 0.29 | 1 | 0 | 0.19 | 0.01 | 0.50 | 1.99 | 5 |
| Heparin hose, EMD-4566 | 1 | -1 | 161.1 | 30.6 | 42.6 | 0.92 | 1 | 0 | 0.62 | 0.18 | 0.54 | 3.26 | 11 |

§. Left-hand twisted filaments are assigned a *hnd* value of -1 and right-hand twisted filaments are assigned a *hnd* value of 1

†. The mean cross-over distance of the selected dGAE fibril shown in **Fig 1c-d**. The overall comparative similarity rank is unchanged if the population mean *cod* value of dGAE fibrils of 72.7 nm (Al‐Hilaly et al., 2020) is used instead.
